# Large Pleomorphic Adenoma of The Upper Lip in a Middle‐Aged Woman With Facial Deformity, Tooth Displacement, and Bone Loss: Uncommon Clinical Presentation of a Benign Tumor

**DOI:** 10.1155/crid/8865055

**Published:** 2026-02-11

**Authors:** Fabiana da Silva de Oliveira, Elias Antonio da Silva Filho, Marcela Adell Trench, Daniele Heguedusch, Fábio Daumas Nunes, Celso Augusto Lemos, Norberto Nobuo Sugaya

**Affiliations:** ^1^ Department of Stomatology, School of Dentistry, University of São Paulo, São Paulo, São Paulo, Brazil, usp.br

**Keywords:** benign mixed tumor, case report, minor salivary gland, pleomorphic adenoma, surgical excision

## Abstract

Pleomorphic adenoma (PA) is the most common benign neoplasm of the salivary glands, occurring in both major and minor glands, with a predilection for the intraoral palate. Its presentation in the upper lip is rare and, when large, may result in facial deformity, alveolar bone resorption, and tooth displacement, especially in long‐standing lesions. Clinically, PA presents as a solid, painless, slow‐growing, and well‐circumscribed mass, which may cause functional morbidity depending on its size and location. The differential diagnosis includes canalicular adenoma, other benign minor salivary gland neoplasms, mesenchymal tumors (lipoma, fibroma, neurofibroma, schwannoma, and hemangioma), and cystic lesions (mucocele, dermoid cyst, and epidermoid cyst). Distinguishing among these entities requires a detailed clinical evaluation, imaging studies, and histopathological confirmation, often complemented by immunohistochemistry. Complete excisional biopsy with adequate margins remains the treatment of choice, providing an excellent prognosis and reducing the risk of recurrence or malignant transformation. This report describes an uncommon case of large PA of the upper lip, associated with alveolar bone loss and tooth displacement, highlighting the importance of early diagnosis and appropriate surgical management.

## 1. Introduction

Pleomorphic adenoma (PA) is the most prevalent benign neoplasm of the salivary glands, accounting for 40%–70% of all tumors affecting both major and minor salivary glands. This neoplasm can develop in different salivary glands, with the palate being the most common intraoral site [1]. PA is a mixed‐origin tumor, comprising epithelial and mesenchymal components, typically enclosed by a fibrous capsule. Its name derives from the architectural pleomorphism observed under light microscopy [2].

Although less common in the upper lip, PA may affect this site occurring in approximately 9.8% of cases [3]. In this location, lesions are generally diagnosed at early stages and treated by surgical excision before causing significant aesthetic deformities, which explains why they are rarely evaluated by otorhinolaryngologists [4]. Furthermore, manifestations such as ulceration, pain, or sensory changes are uncommon [5]. Despite its benign nature, the tumor may extend into surrounding glandular tissue through digitiform projections (pseudopodia), contributing to recurrences after excision [6].

The differential diagnosis of upper lip swelling includes cystic lesions (mucocele, dermoid cyst, and epidermoid cyst), benign tumors (fibroma, hemangioma, lipoma, and schwannoma), and malignant tumors (squamous cell carcinoma, mucoepidermoid carcinoma, and adenoid cystic carcinoma), as well as other conditions such as foreign body reaction, infection, orofacial granulomatosis, Quincke edema, tuberculosis, and actinomycosis [7]. Canalicular adenoma should also be considered in the differential diagnosis, particularly in cases of upper lip enlargement. It is a rare, benign epithelial neoplasm of minor salivary glands, clinically presenting as a painless, slow‐growing, usually solitary submucosal nodule, with a predilection for the upper lip (approximately 80% of cases). Diagnosis is confirmed by histopathological and immunohistochemical examination [8].

Depending on the size and location of the tumor, large lesions can compromise functions such as breathing, swallowing, and speech, leading to significant morbidity and even potential mortality [9]. Additionally, progressively growing tumors may cause marked facial alterations, impairing both the form and function of the structures involved.

Furthermore, PA has the potential for malignant transformation into carcinoma ex‐pleomorphic adenoma (CXPA), carcinosarcoma, or metastatic PA, with CXPA being the most common form. Its prevalence is estimated at 1.5% within the first 5 years after diagnosis, reaching up to 10% after 15 years; in recurrent cases, the rate is approximately 3.3% [10].

In rare instances, PA of the lip may lead to alveolar bone resorption and tooth displacement, particularly when the tumor is large or long‐standing, resulting in significant aesthetic and functional alterations. Diagnostic confirmation requires imaging studies and biopsy, with complete surgical resection remaining the treatment of choice. Histologically, PA is characterized by epithelial cells arranged in cords, areas of squamous differentiation, and plasmacytoid myoepithelial cells, which produce an abundant extracellular matrix composed of chondroid, collagenous, myxoid, and osseous stroma [11].

In this context, we report an unusual case of a large PA of the upper lip, associated with alveolar bone resorption in the region of Tooth 21, resulting in tooth displacement and mobility, as well as facial deformity, features rarely described at this location.

## 2. Case Report

**Figure 1:**
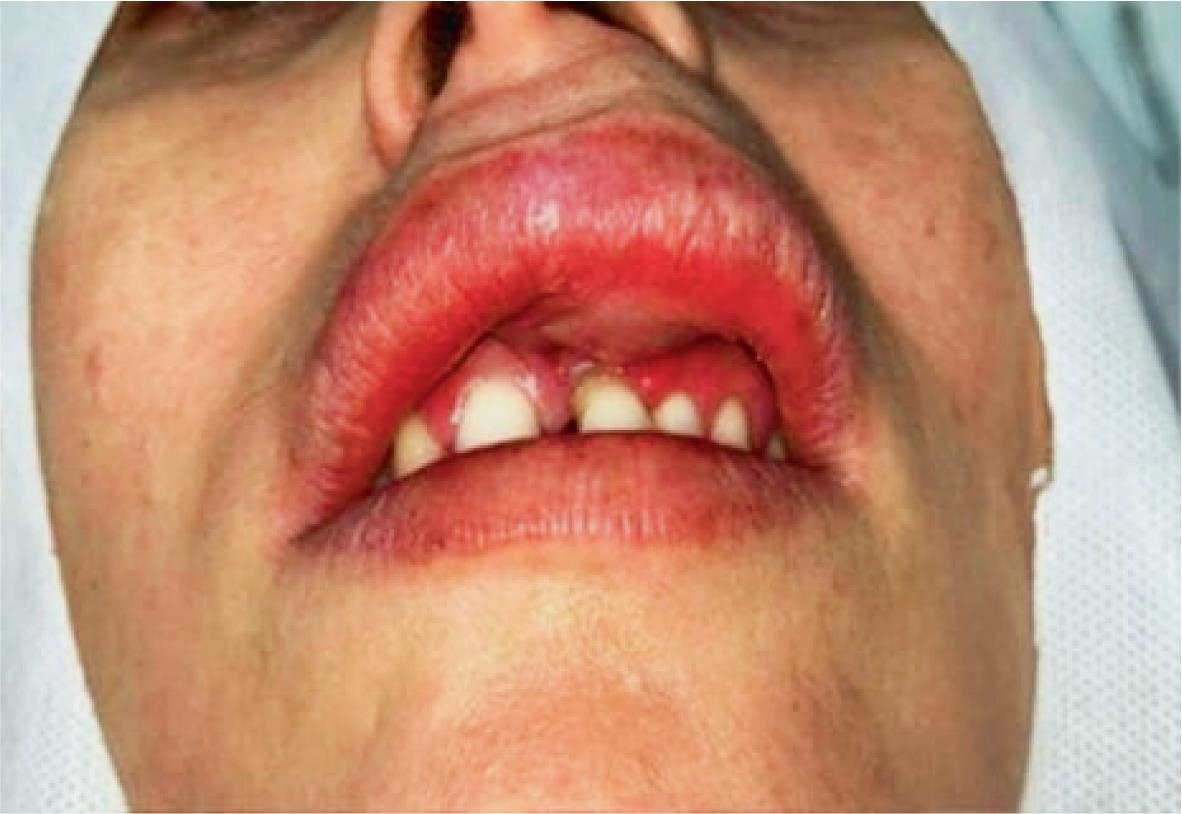
Initial clinical aspect showing swelling of the left upper lip, with noticeable facial asymmetry, elevation of the ipsilateral nasal ala, and slight pressure exerted on the left maxillary central incisor.

A 41‐year‐old White woman was referred to the Oral Medicine Clinic by her private dentist for evaluation of a swelling in the upper lip, present for approximately 3 years. The patient reported having sustained trauma to that region during childhood; however, the recent and progressive onset of the swelling and the development of facial asymmetry indicated that the condition was not related to the remote traumatic event, but rather to an evolving neoplasm. The nodule began to develop on the left upper labial mucosa 3 years earlier and showed progressive growth (Figure 1).

The patient was not taking any medications, and a medical evaluation conducted 1 year earlier revealed no associated systemic conditions. Extraoral examination demonstrated noticeable facial asymmetry, with edema of the upper lip and slight displacement of the left nostril. On palpation, a single, firm, nontender nodule was identified, not adherent to the underlying bone or overlying skin.

**Figure 2:**
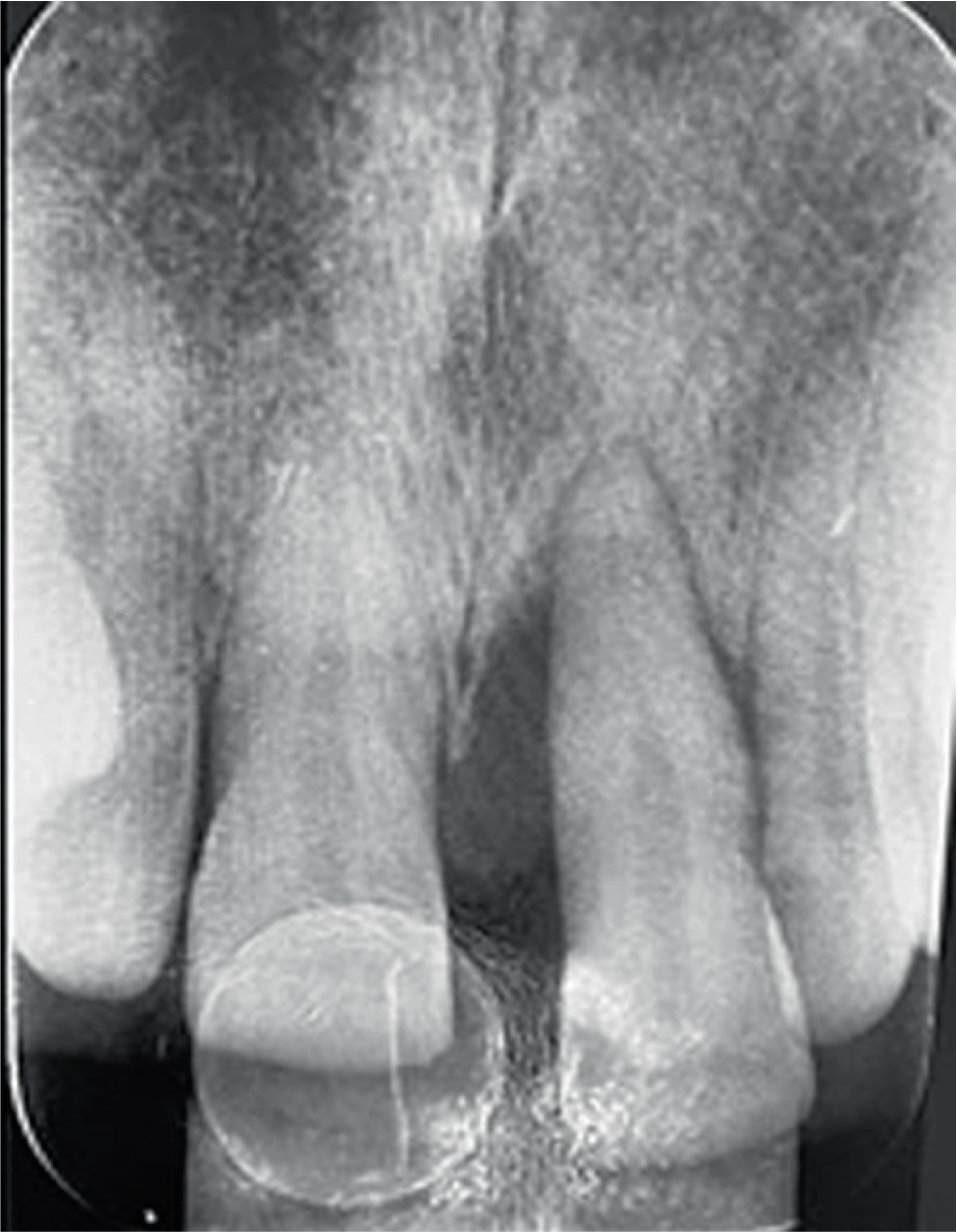
Periapical radiograph showing alveolar bone loss on the mesial surface of the left maxillary central incisor, with slight root displacement but no evidence of internal resorption or periapical changes.

Periapical radiography revealed significant alveolar bone loss at the mesial aspect of the left maxillary central incisor (Figure 2), although the tooth remained vital and stable.

**Figure 3:**
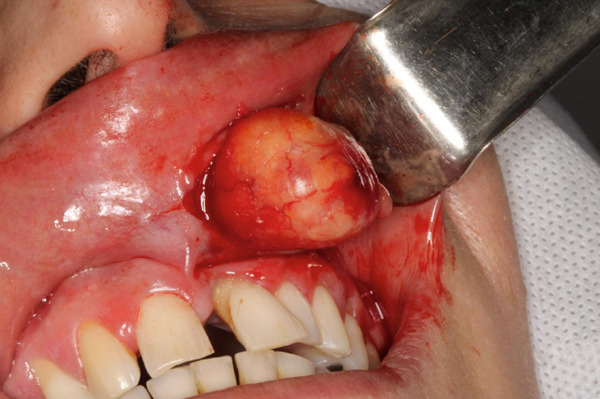
Intraoperative view showing a well‐defined, encapsulated nodular lesion with a whitish‐reddish surface, being removed by blunt dissection from the left upper labial mucosa.

The clinical differential diagnosis included PA and canalicular adenoma. In the absence of surgical contraindications, complete excision of the lesion was performed. Preoperative fine‐needle aspiration confirmed the solid nature of the tumor, and the procedure was carried out under local anesthesia, with incision and total surgical removal of the lesion (Figure 3).

Histopathological analysis revealed a classic PA architecture, characterized by epithelial cells arranged in cords and islands, interspersed with plasmacytoid myoepithelial cells, and a prominent myxoid and chondroid extracellular matrix (Figure 4).

Figure 4(a) Histological section showing a fragment of salivary gland neoplasm, characterized by the proliferation of myoepithelial cells with epithelioid or plasmacytoid morphology (40x). (b) The cells form numerous duct‐like spaces composed of two layers of cells. (c) Neoplastic cells displaying extensive areas of chondroid metaplasia (100x). (d) Formation of duct‐like structures of varying sizes, with an inner layer of cuboidal epithelial cells and an outer layer of spindle‐shaped myoepithelial cells.

**Figure:**
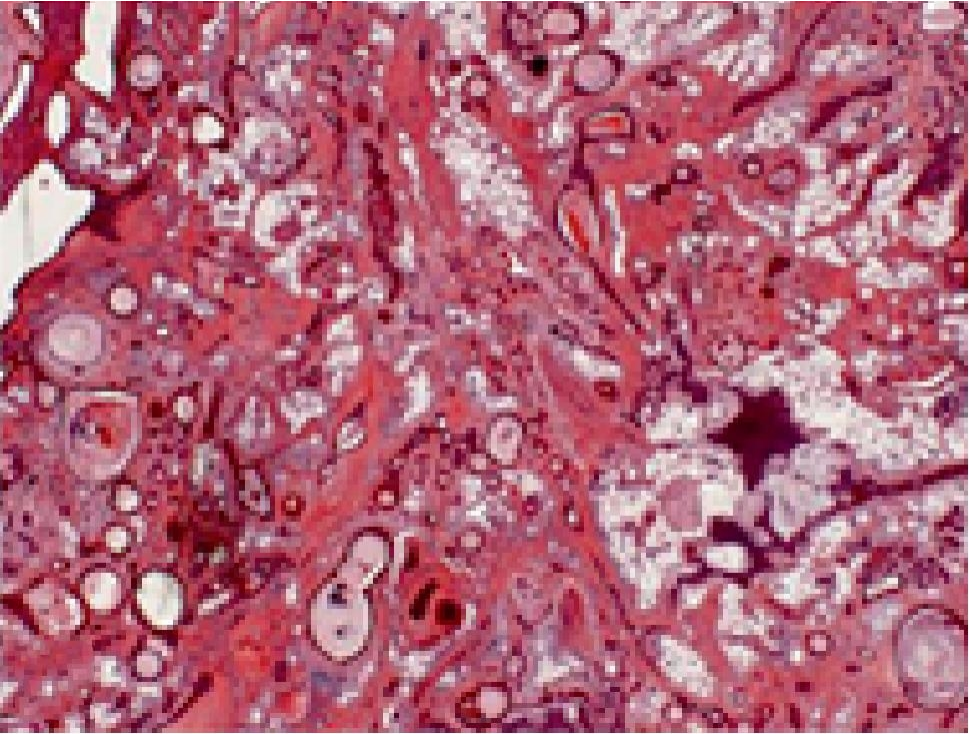
(a)

**Figure:**
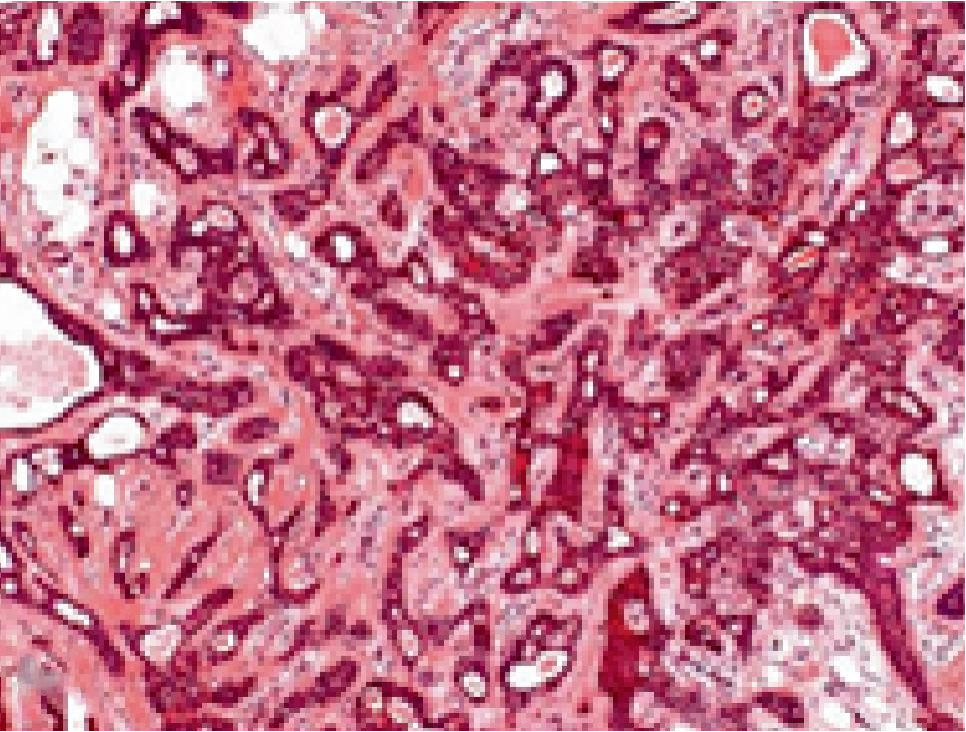
(b)

**Figure:**
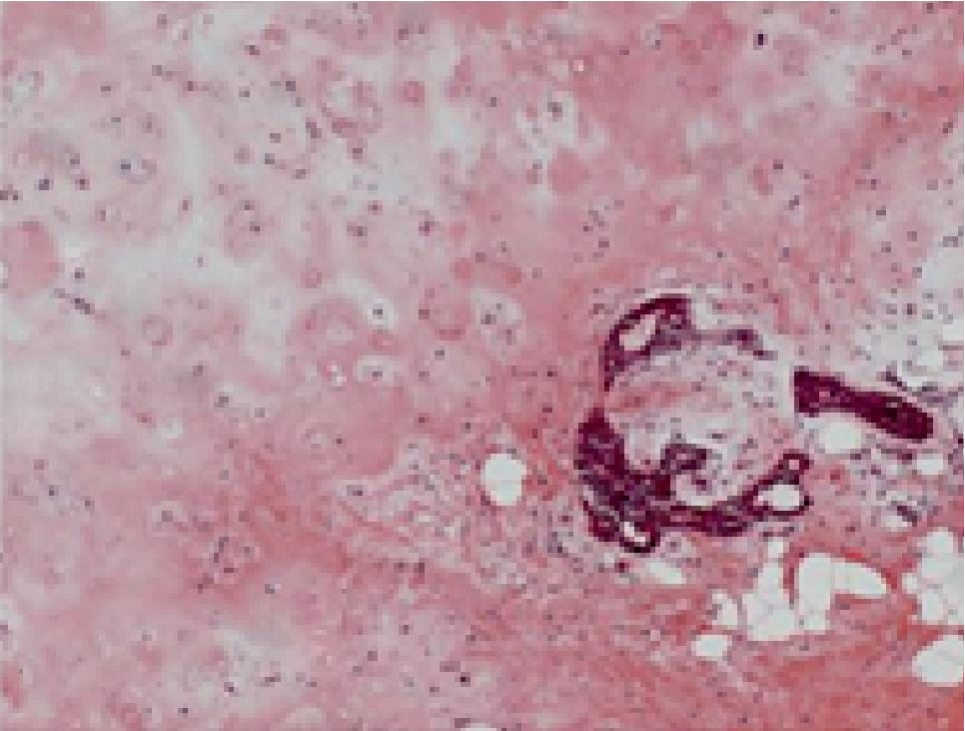
(c)

**Figure:**
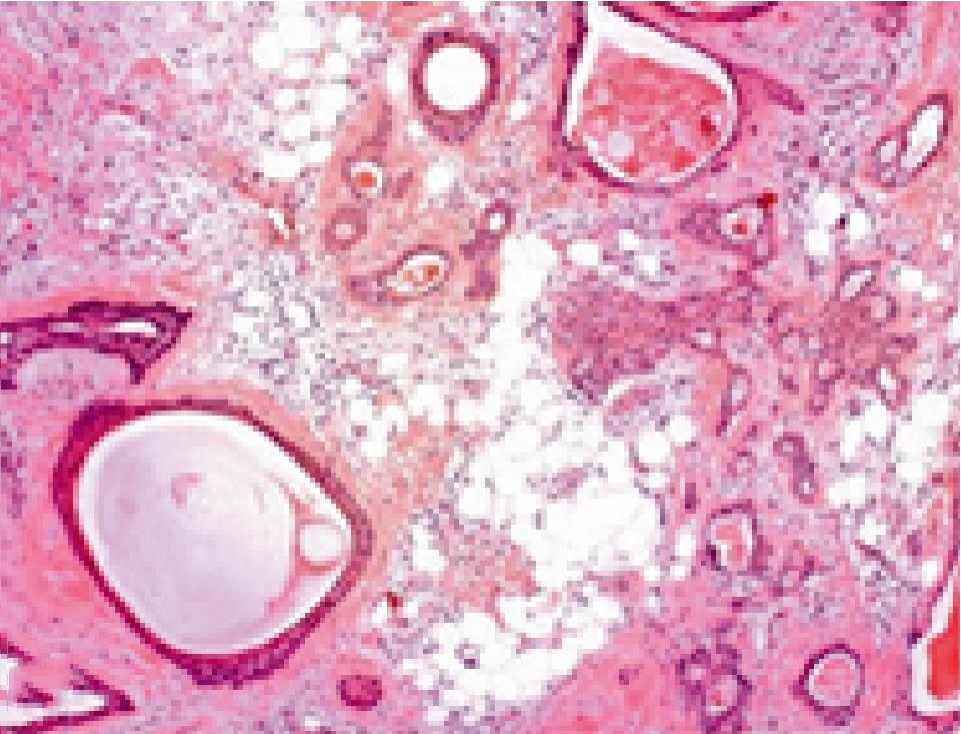
(d)

The fibrous capsule was intact, with no evidence of capsular invasion, supporting the benign diagnosis and emphasizing the importance of complete surgical excision. These histological features corresponded to the firm consistency observed on palpation and the slow yet expansive growth, which accounted for the tooth displacement and alveolar bone resorption.

One week postoperatively, the patient demonstrated uneventful recovery, with no hematomas or complaints. The excised tumor appeared as an ovoid, well‐circumscribed, fully encapsulated mass, white to reddish in color, with a solid cut surface, measuring approximately 3 cm in diameter. At 1‐year follow‐up, the mucosa of the left upper lip remained intact, with no signs of recurrence.

## 3. Discussion

PA is a mixed salivary gland neoplasm characterized by marked histological heterogeneity, with its morphological diversity justifying the designation “pleomorphic” [12]. Clinically, it presents as a firm, painless, slow‐growing mass, generally mobile when small, but it may become fixed to adjacent tissues in advanced stages. Prognosis is favorable when the tumor is completely removed, with wide and complete surgical excision being the treatment of choice. However, late recurrences and malignant transformation are possible, making long‐term clinical follow‐up necessary [13].

Recurrences are associated with significant complications, such as an increased risk of facial nerve paralysis and further recurrences after revision surgeries, as well as the possibility of malignant transformation. Malignant transformation occurs in less than 5% of cases and is related to the progressive accumulation of genomic alterations [14, 15]. Major risk factors include incomplete excision, intraoperative capsular rupture, myxoid subtype, presence of satellite nodules, pseudopodia, absence of glandular tissue margins, and surgeon experience. Other possible contributing factors are female sex, young age, tumor location, size, and duration of lesion evolution [16].

Malignant transformation of PA is a rare phenomenon, and its hypothesis is primarily supported by the identification of residual areas of PA in malignant tumors, which are therefore classified as CXPA. In these cases, a gradual morphological and molecular transition is observed between the benign PA component and the malignant component [17].

Risk factors associated with malignant transformation include age over 50 years, history of smoking, and tumors larger than 2 cm in diameter. Clinical signs suggestive of malignancy, such as rapid growth or pain, may be absent, emphasizing the importance of careful clinical and histopathological follow‐up [18].

CXPA exhibits aggressive behavior, with an unfavorable prognosis and an estimated malignancy rate of approximately 6.2%. The 5‐year survival rate ranges from 25% to 65%, with frequent lymph node metastases and disease‐related deaths. Although cancer stem cells (CSCs) are recognized as central elements in carcinogenesis, their specific role in the development and progression of CXPA remains incompletely elucidated [19].

The average size of most intraoral mixed tumors is less than 3.0 cm [20]. The patient in this study was a 41‐year‐old young adult who did not smoke or consume alcohol. The lesion submitted to excision measured approximately 3 cm in diameter, representing a relatively large lesion, but it had a complete and intact capsule, without rupture, a situation considered favorable for reducing the risk of malignancy. Despite a duration of about 3 years, the presence of facial deformity prompted the patient to seek care. The surgical excision was performed completely, which significantly contributes to a positive prognosis and reduces the likelihood of recurrence.

The alveolar bone is the portion of the maxilla responsible for supporting the teeth via the periodontal ligament, acting in the absorption and distribution of occlusal forces during mastication. Due to its complex formation, it has a low regenerative capacity after tooth loss, and its remodeling depends on osteoclastic activity. Mechanical stress plays a crucial role both in the maintenance and pathogenesis of periodontal alterations, directly influencing the structural integrity of this region [21, 22].

Pathological tooth migration occurs when there is a change in the position of teeth due to the interruption of the forces that maintain them in the dental arch. Etiological factors include loss of the periodontal attachment apparatus and nonperiodontal conditions, such as excessive force, which can lead to progressive tooth movement and perceptible aesthetic changes in the smile line [23].

Alveolar resorption observed in cases of large PA of the lip can be explained by chronic compression exerted over adjacent teeth. Although the tumor is benign, its slow and expansive growth can generate continuous pressure on the alveolar bone and tooth roots, promoting gradual bone tissue loss without the need for direct periodontal ligament inflammation. This secondary effect, although rare, can result in localized bone resorption in addition to contributing to facial deformity and functional impairment, highlighting the importance of early diagnosis and complete surgical excision of the tumor.

The differential diagnosis of a large upper lip mass should consider a wide variety of lesions, including minor salivary gland tumors and mesenchymal neoplasms. Among benign salivary gland tumors, PA and canalicular adenoma stand out, both slow‐growing, well‐circumscribed, and generally painless, with the latter showing a higher predilection for the upper lip [24, 25].

Mesenchymal neoplasms, such as lipoma, fibroma, neurofibroma, schwannoma, and hemangioma, should also be included in the differential diagnosis, as they may present similar clinical features, varying in consistency, mobility, and coloration. In addition, cystic lesions, such as mucocele, dermoid cyst, and epidermoid cyst, which may occur in the same region, should be considered during clinical evaluation [26].

Differentiation among these entities is based on clinical and imaging findings, with the definitive diagnosis established by histopathological examination, sometimes complemented by immunohistochemical analysis. Table 1 summarizes the main lesions associated with upper lip swelling, highlighting their nature, clinical course, and potential to cause facial deformity.

**Table 1:** Comparison of the main cystic lesions and benign tumors causing upper lip swelling, highlighting their nature, clinical course, and potential to cause facial deformity.

Lesion/condition	Nature	Clinical course	Potential for facial deformity
Mucocele	Benign cyst	Recurrent, self‐limiting	Low
Dermoid cyst	Benign cyst	Slow‐growing	Moderate
Epidermoid cyst	Benign cyst	Similar to dermoid	Moderate
Fibroma	Benign tumor	Firm, slow‐growing	Low
Hemangioma	Benign vascular tumor	May grow rapidly	Moderate to high
Lipoma	Benign adipose tumor	Slow‐growing	Moderate
Schwannoma	Benign neural tumor	Encapsulated, persistent	Moderate
Neurofibroma	Benign neural tumor	Nonencapsulated, progressive growth	Moderate to high
Canalicular adenoma	Benign minor salivary gland tumor	Slow‐growing, usually painless	Moderate
Pleomorphic adenoma	Benign minor salivary gland tumor	Slow‐growing, may reach large size	Moderate to high

*Source:* The authors.

Although the long‐standing solid mass suggests benignity, comprehensive clinical and histopathological evaluation is essential to avoid misdiagnosis. As these tumors are generally encapsulated, the treatment of choice is complete excisional biopsy with adequate margins [27].

In this case, the significant tumor volume, associated with facial deformity and bone resorption, represented an unusual presentation of PA of the upper lip, requiring the exclusion of mesenchymal and cystic lesions with more aggressive behavior.

## 4. Conclusion

PA of the upper lip, although benign, may cause bone resorption and facial deformity when it reaches large dimensions. The differential diagnosis should include canalicular adenoma, other salivary gland neoplasms, as well as mesenchymal or cystic lesions. Complete surgical excision with adequate margins is the treatment of choice, providing an excellent prognosis and minimizing the risk of recurrence or malignant transformation, highlighting the importance of early diagnosis and careful management.

## Funding

This paper was financed in part by the Coordenação de Aperfeiçoamento de Pessoal de Nível Superior–Brasil (CAPES)–Finance Code 001.

## Consent

Written informed consent has been received from the patients.

## Conflicts of Interest

The authors declare no conflicts of interest.

## Data Availability

The data that support the findings of this study are available from the corresponding author upon reasonable request.
